# Dynamic Construction of Reduced Representations in the Brain for Perceptual Decision Behavior

**DOI:** 10.1016/j.cub.2018.11.049

**Published:** 2019-01-21

**Authors:** Jiayu Zhan, Robin A.A. Ince, Nicola van Rijsbergen, Philippe G. Schyns

**Affiliations:** 1 Institute of Neuroscience and Psychology, University of Glasgow, Scotland G12 8QB, United Kingdom; 2 School of Psychology, University of Glasgow, 62 Hillhead Street, Glasgow, Scotland G12 8QB, United Kingdom

**Keywords:** reverse correlation, MEG, neural networks, ambiguous perception, information processing, data reduction, decision making, SIR, internal representations, brain dynamics

## Abstract

Over the past decade, extensive studies of the brain regions that support face, object, and scene recognition suggest that these regions have a hierarchically organized architecture that spans the occipital and temporal lobes [1, 2, 3, 4, 5, 6, 7, 8, 9, 10, 11, 12, 13, 14], where visual categorizations unfold over the first 250 ms of processing [15, 16, 17, 18, 19]. This same architecture is flexibly involved in multiple tasks that require task-specific representations—e.g. categorizing the same object as “a car” or “a Porsche.” While we partly understand where and when these categorizations happen in the occipito-ventral pathway, the next challenge is to unravel how these categorizations happen. That is, how does high-dimensional input collapse in the occipito-ventral pathway to become low dimensional representations that guide behavior? To address this, we investigated what information the brain processes in a visual perception task and visualized the dynamic representation of this information in brain activity. To do so, we developed stimulus information representation (SIR), an information theoretic framework, to tease apart stimulus information that supports behavior from that which does not. We then tracked the dynamic representations of both in magneto-encephalographic (MEG) activity. Using SIR, we demonstrate that a rapid (∼170 ms) reduction of behaviorally irrelevant information occurs in the occipital cortex and that representations of the information that supports distinct behaviors are constructed in the right fusiform gyrus (rFG). Our results thus highlight how SIR can be used to investigate the component processes of the brain by considering interactions between three variables (stimulus information, brain activity, behavior), rather than just two, as is the current norm.

## Highlights

• We show how the brain reduces high-dimensional input into decision representations
• Occipital cortex reduces features irrelevant for behavior ∼170 ms post stimulus
• Past 170 ms, fusiform gyrus combines the features supporting behavioral decisions

## Teaser

In a decision task, Zhan et al. visualize within a new information theoretic framework the dynamic representation of visual information in brain activity. They demonstrate rapid reduction of behaviorally irrelevant information in the occipital cortex and a combination of the features that supports distinct decisions in the right fusiform gyrus.

## Results

### Diagnostic Features of Behavior: Identifying the Stimulus Features that Underlie Perceptual Decisions

**Figure 1:**
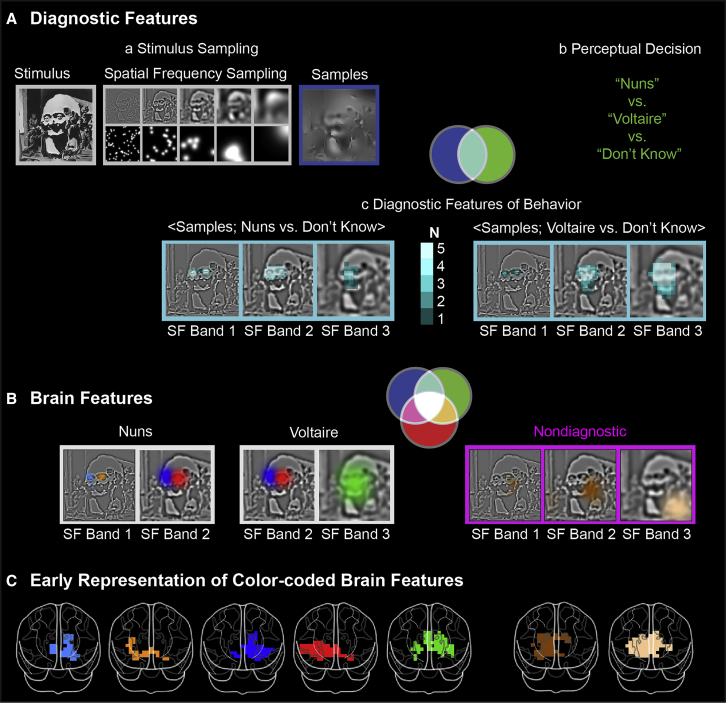
Diagnostic and Brain Features (A) Diagnostic features. (a) The original stimulus (left), which was decomposed into 6 spatial frequency (SF) bands (middle, band 6 is not shown) of one octave each for each trial, starting at 128 cycles per image. Samples were added across bands to generate one experimental stimulus (dark blue frame, right). (b) Perceptual decisions recorded by observers, as: “the nuns,” “Voltaire,” or “don’t know.” The cyan intersection in the Venn diagram illustrates the relationship between information samples (blue) and perceptual decisions (green): the diagnostic features of behavior. (c) Diagnostic feature of behavior. The cyan-framed images show significant pixels (family-wise error rate (FWER), p < 0.001, one-tailed) in the first three SF bands that reveal features diagnostic for observers responding “the nuns” (the two small faces in SF band 1) and “Voltaire” (the broad face in SF band 3). Color saturation indicates N, number of observers. (B) Brain features. White frames highlight “the nuns” and “Voltaire” diagnostic and color-coded brain features represented by all observers. The magenta frames highlight color-coded non-diagnostic brain features represented by a majority of observers (i.e., N > = 3). The magenta intersection in the Venn diagram represents the relationship between information samples (blue) and MEG voxel activity (red) whereas the white intersection represents the relationship between all three variables, including behavior. (C) Early representation of brain features. Common, color-coded brain regions, show the early (during the initial 20 ms of representation) topological representation of each correspondingly colored brain feature (FWER, p < 0.05, one-tailed). Each observer contributed at least one significant voxel for each color-coded feature. See also Figure S1 for the results of each observer.

In this task, we used Dali’s painting *Slave Market with Disappearing Bust of Voltaire* (see Figure 1A-a, Stimulus) because it contains a complex, ambiguous scene that observers perceive as either “the nuns” or “Voltaire.” We used the Bubbles technique [20] to break down the stimulus information into random samples for each experimental trial (see Figure 1A-a, Stimulus Sampling) to characterize the features that support each perceptual decision. We then recorded the observer’s response to each sample (whether they perceived “the nuns,” “Voltaire,” or selected “don’t know”) and also their dynamic brain activity (on 12,773 MEG sources, every 2 ms between 0 and 400 ms post-stimulus, see STAR Methods). Using this approach, we identified the low-dimensional information each participant used to support their “nuns” versus “Voltaire” decision by evaluating the relationship between the randomly sampled information on each trial and the corresponding observer’s decision (see Figure 1A-b). We schematized the relationship between the two variables of information sample and decision as a Venn diagram; where they intersect was designated the “diagnostic features” that support each observer’s decisions (Figure 1A) [21, 22]. Specifically, using mutual information (MI [23], a nonparametric measure of the relationship between variables), we computed diagnostic features separately for the behavioral contrasts <Information Samples; “the nuns,” versus “don’t know”>, excluding “Voltaire” trials, and <Information Samples; “Voltaire,” versus “don’t know”>, excluding “nuns” trials (see STAR Methods).

As shown in Figure 1A-c, all observers used the left and right nun’s faces at higher spatial frequencies (HSF) to respond “the nuns,” whereas they used the global face of Voltaire at lower spatial frequencies (LSF) to respond “Voltaire” (see Figure S1-A for each observer’s features). Since diagnostic features influence behavior, the observer’s brain must represent at a minimum these features between stimulus onset and observer decision. Next, we show that the brain does indeed represent all diagnostic features over time, as well as other features.

### Sampled Information Coupled to MEG Voxel Activity: Characterizing the Stimulus Features that Brain Activity Represents

To show where and when each observer’s MEG activity represents stimulus features, we used MI to evaluate the single-trial relationship <Information Samples; MEG Source Activity> independently for each source (henceforth, MEG voxel) and time point (see STAR Methods). The outcome is a 3D (feature-by-voxel-by-time) MI matrix per observer in which, for each stimulus feature represented in their brain (1st dimension), MI values indicate the strength of feature representation (i.e., effect size, FWER p < 0.05, one-tailed) over 12,773 MEG voxels (2nd dimension), every 2 ms between 0 and 400 ms post-stimulus (3rd dimension). These 3D representation matrices are unique to our approach: they reveal the stimulus features that the brain dynamically represents, separating out the features that are relevant for the perceptual task.

First, we identified the features represented in observers’ brains (see Figure 1B for the common features represented cross observers and Figure S1-B for each observer’s brain features). Comparing Figure 1B with Figure 1A reveals that some brain features correspond to the same visual information as the features that are diagnostic of behavior (i.e., the red and blue nun’s faces at HSFs and the green face of Voltaire at LSFs), whereas others do not (e.g., the brown features flanking Voltaire’s face).

Second, we divided the brain’s features into diagnostic or nondiagnostic for the task (see STAR Methods). The Venn diagram of Figure 1B illustrates such division: the addition of brain measures produces a white area of intersection that represents the diagnostic features that influence both behavioral and brain measures; the magenta intersection designates the nondiagnostic features that influence brain measures but not behavior. (see Figure S1C and Figure S2A for a formal demonstration).

Finally, Figure 1C illustrates the expected topological representation of brain features during the first 20 ms of representation (see Figure S1F for each observer’s topological representation). Color codes reveal that the observers’ brains contral-laterally represented the diagnostic eyes of Voltaire (see the red and blue voxels) and the brown nondiagnostic features flanking the center of the stimulus in relation to the bilaterally represented LSF Voltaire face (see green voxels).

### Divergence of Nondiagnostic and Diagnostic Brain Features in the Occipito-Ventral Pathway

**Figure 2:**
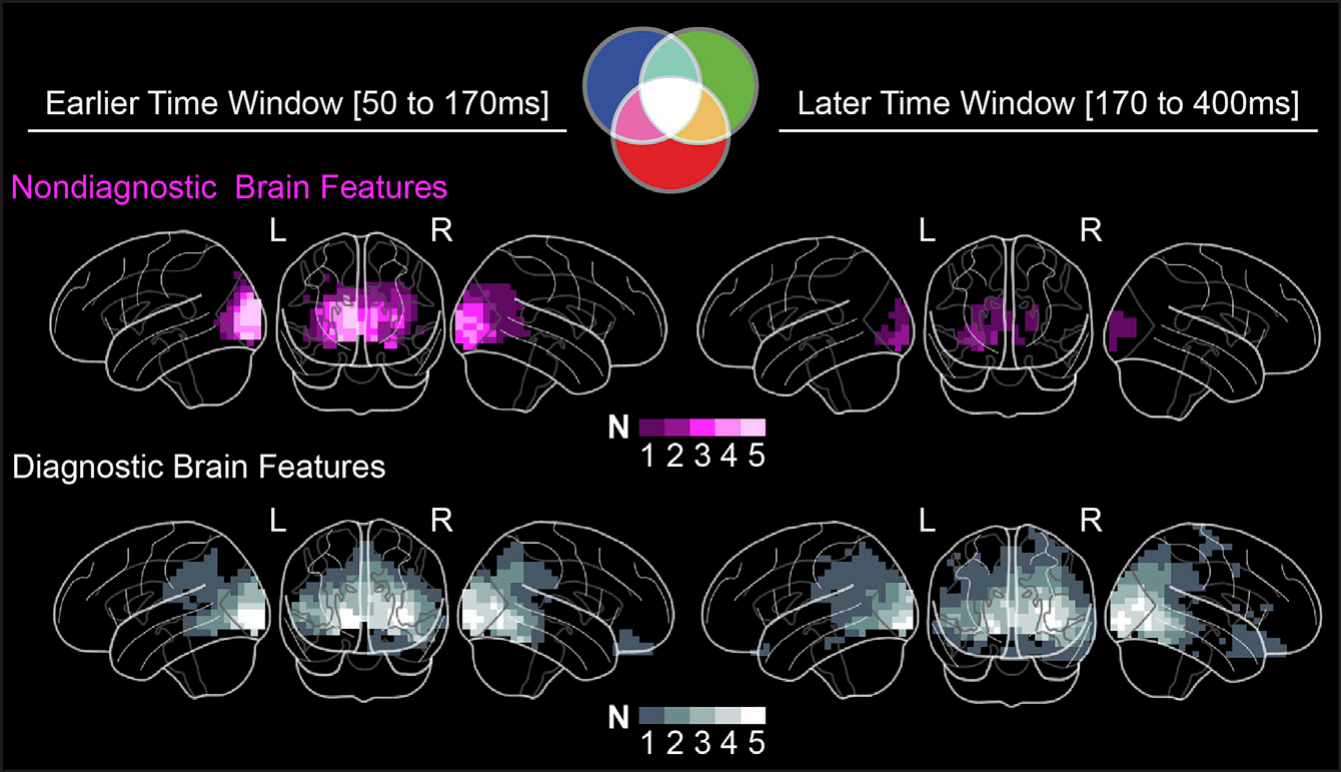
Nondiagnostic Feature Reduction and Diagnostic Feature Progression Magenta color-coded brains show voxels that represent at least one significant (FWER p < 0.05, one-tailed) nondiagnostic brain feature (represented with a magenta color in the Venn diagram) in earlier [50–170 ms] and later [170–400 ms] time windows post stimulus. White color-coded brains show voxels that represent at least one significant (FWER p < 0.05, one-tailed) diagnostic brain feature (represented with a white color in the Venn diagram) in earlier [50–170 ms] and later [170–400 ms] time windows post stimulus. Voxel brightness denotes the number (N) of observers for whom these criteria held true. For all observers, nondiagnostic features were consistently reduced over time in the occipital cortex while diagnostic features were sustained and progressed into the ventral pathway. See also Figure S2B. Abbreviations: left (L); right (R).

The color-coded brains in Figure 2 summarize the evolving representations of the diagnostic and nondiagnostic features across two post-stimulus time windows [50–170 ms] and [170–400 ms] that flank the N/M170, the event-related potential ∼170 ms post-stimulus commonly associated with visual categorizations [16, 24]. A comparison of the nondiagnostic and diagnostic brain features across the earlier and later time windows reveals a consistent pattern. Over the first 170 ms of processing, representation of diagnostic and nondiagnostic brain features similarly involve occipital cortex (Bonferroni corrected p < 0.05, two-tailed). They diverge afterward, and only representations of diagnostic brain features are sustained in all occipito-ventral regions (see STAR Methods and Figure S2B for representation divergence in each observer).

These data suggest that a spatio-temporal junction exists between the occipital and occipito-ventral cortex around 170 ms, after which only behaviorally relevant features flow into the temporal cortex, with the processing of irrelevant features ending in the occipital cortex. In the next two sections, we detail what happens before and after this junction.

### Dynamic Reduction of Nondiagnostic Brain Features in the Occipito-Ventral Pathway

**Figure 3:**
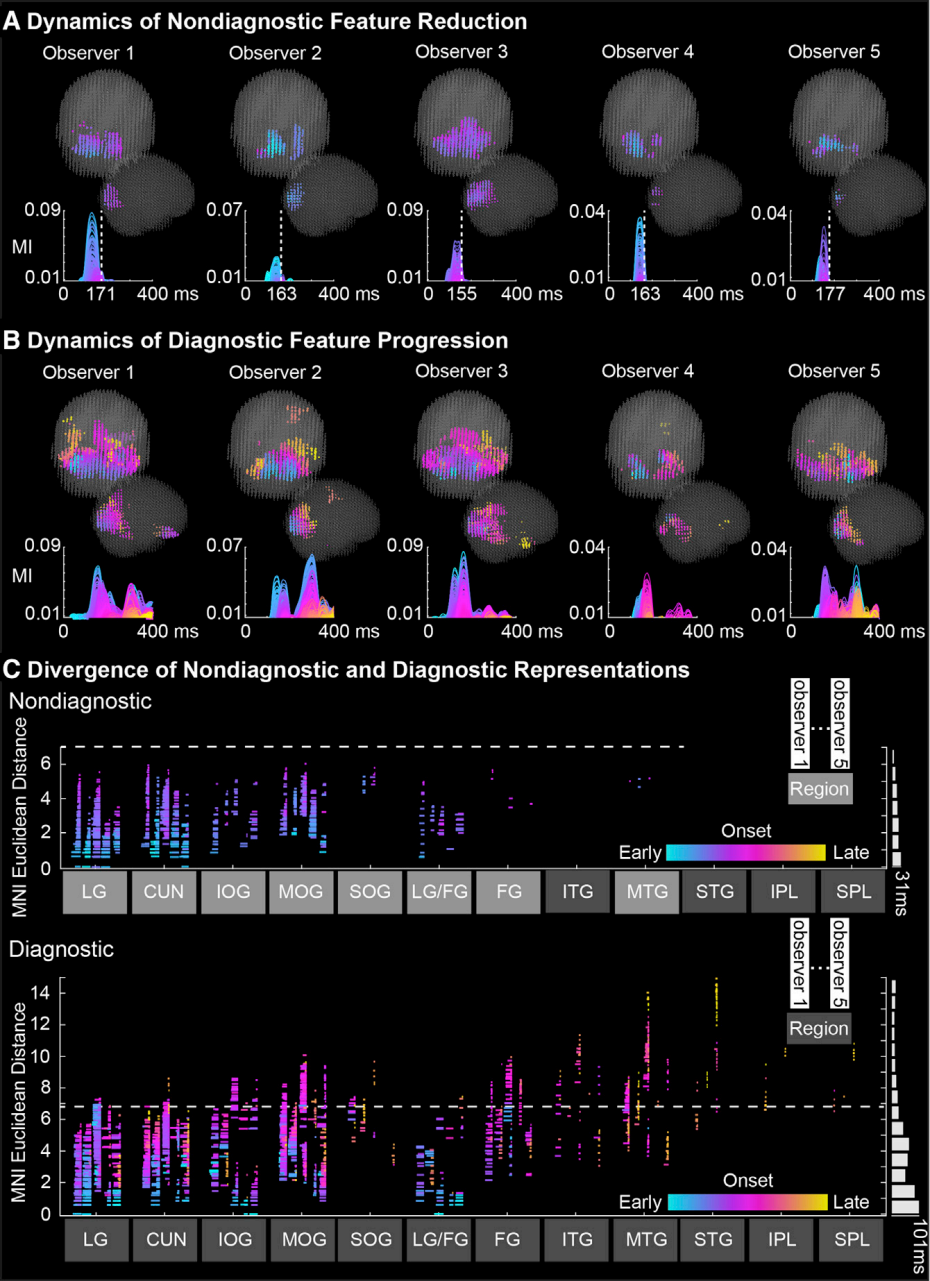
Dynamic Reduction of Nondiagnostic Brain Features in the Occipital-Ventral Pathway. (A and B) Dynamics of (A) nondiagnostic brain feature reduction and (B) diagnostic brain feature progression. For each observer, a plot shows the curves of maximum (A) nondiagnostic and (B) diagnostic brain feature representation (i.e., MI effect size) for each voxel between 0 and 400ms post stimulus, color-coded by ranked onset time (blue, early; magenta, midway; yellow, late). In (A), the vertical dashed lines represent the time (∼170 ms) at which the brain stops representing nondiagnostic features. Adjacent brain scatters locate the voxels associated with each curve using the same color code. (C) Divergence of nondiagnostic and diagnostic feature representations. In each panel, brain regions comprise one column per observer, where each horizontal line represents one voxel from the region. Lines denote two voxel properties: the color denotes representation onset, and the length, representation duration. Adjacent white bars show median representation duration across all regions, organized by the y axis of MNI Euclidean distance of each voxel to the voxel of initial representation onset. The dashed white horizontal line shows the nondiagnostic wavefront extends ventrally in the LG up to the junction with the TG and FG, and dorsally with IPL and SPL (see regions shaded a lighter gray). The diagnostic wavefront extends further into the ventral (i.e., FG, ITG, MTG, and STG) and dorsal (i.e., IPL and SPL) (see pink to yellow colors). See also Figure S3, Video S1 and Table S2. Abbreviations: Cuneus (CU), lingual gyrus (LG), inferior occipital cortex (IOG), middle occipital gyrus (MOG), superior occipital gyrus (SOG), fusiform gyrus (FG), inferior temporal gyrus (ITG), middle temporal gyrus (MTG), superior temporal gyrus (STG), inferior parietal lobe (IPL), and superior parietal lobe (SPL).

Diagnostic and nondiagnostic features travel like two wavefronts of representation in the occipital cortex toward the occipito-ventral junction, where they diverge ∼170 ms post stimulus. To establish this finding, we used each observer’s 3D representation matrix and computed the maximum representation strength (i.e., MI effect size) across nondiagnostic (versus diagnostic) brain features separately for each voxel and time point (see STAR Methods). This produced a time course of maximum feature representation (see Figures 3A and 3B).

In Figure 3A, the representation time courses and brain scatters illustrate the dynamic reduction of nondiagnostic feature representations in each observer (see Video S1 for the dynamic effects in a typical observer). Specifically, nondiagnostic feature representations initially travel as a wavefront that then reduces in duration as it progresses through the occipital cortex (see Figure S3A for each observer and Table S2 for demonstrations). Thus, the wavefront of nondiagnostic feature representations rapidly collapses (around 170 ms) as it travels into the occipital cortex (see Figure 3A). In contrast, identical computations applied to diagnostic features demonstrate that the diagnostic wavefront progresses past 170 ms and deeper into ventral and dorsal regions (see Figures 3B and S3B for each observer and Table S2 for demonstrations). We also identified the anatomical brain regions where the two wavefronts diverge (see Figures 3C and S3C for each observer).

Video S1. Location of Voxels Representing Diagnostic and Nondiagnostic Brain Features at Each 4 ms Time Point between 0 and 400 ms of Observer 1, Related to Figure 3 The upper row shows the diagnostic representation and the lower row shows the nondiagnostic representation. A cyan-to-yellow color represents the earlier to later onset time of each feature representation at each voxel.

### Dynamic Construction of Behavior Representations in the Right Fusiform Gyrus

Our results show that only diagnostic brain features are represented past the occipito-ventral 170 ms junction. A prevalent hypothesis is that visual information represented early and separately across the left and right occipital cortices [25] later converges in the rFG to support visual cognition tasks, such as visual decisions [26]. However, conclusive testing of this hypothesis remains challenging for two reasons. First, the hypothesis implies the need to characterize the evolution of increasingly complex (e.g., lateralized to bilateral) stimulus representations in the dynamic brain activity of this specific region, and not others. Second, it requires demonstrating that the representations specifically support task behaviors.

We propose that the SIR framework can address these points in a data-driven manner across the whole brain. We introduce feature redundancy (RED), which quantifies the shared variability between: < Information Samples; MEG Voxel Activity; “the nuns,” “Voltaire,” “don’t know”> on individual trials. It therefore directly measures modulations of feature representations in the brain to specifically support each perception. We computed feature redundancy (FWER p < 0.05, one-tailed) on all 12,773 MEG voxels of each observer over an extended N/M170, 120–220 ms post-stimulus time course [19, 24] (see STAR Methods).

**Figure 4:**
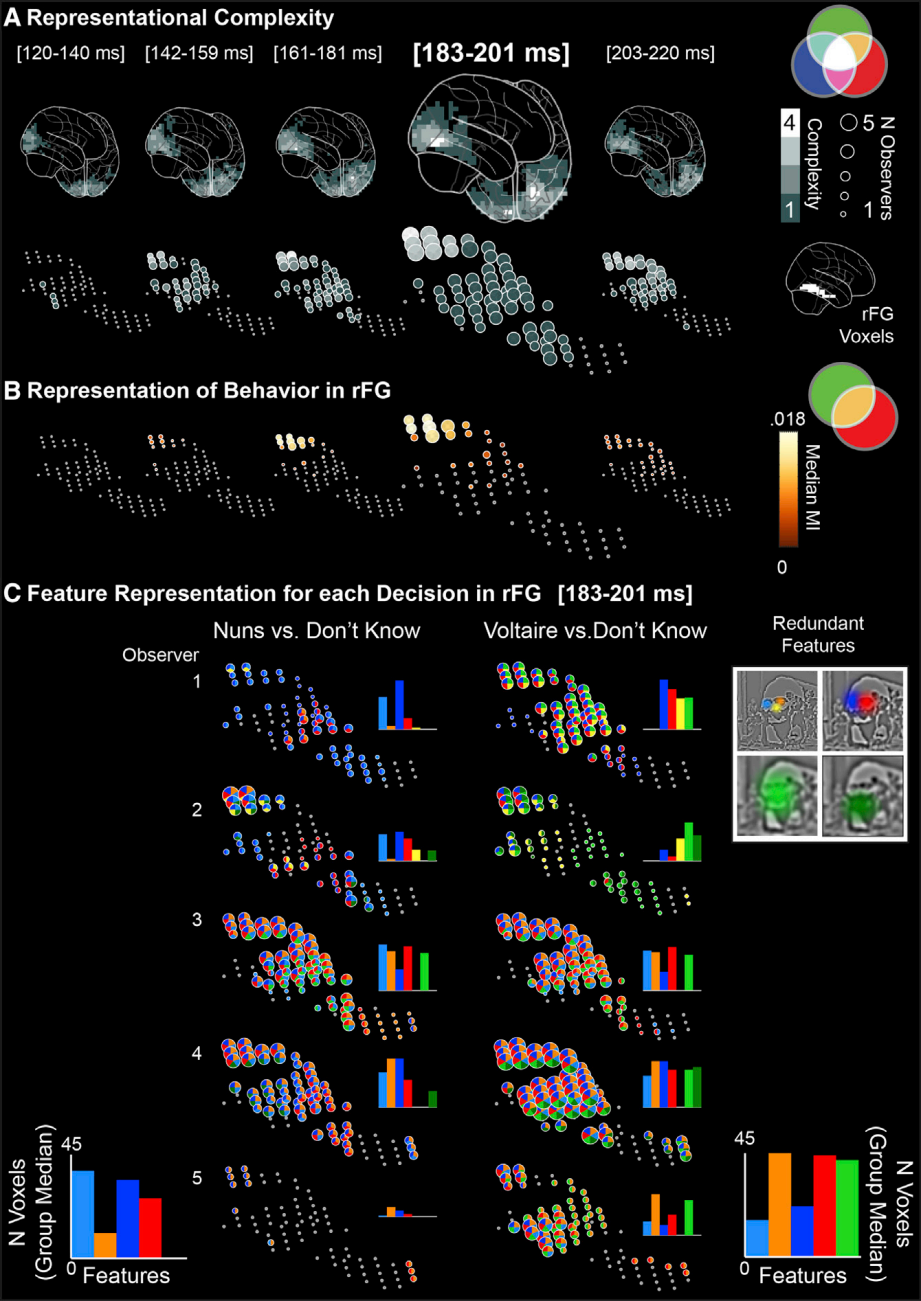
Dynamic Construction of Behavior Representations (A) Representational complexity. Gray level voxels in each brain schematic and in each time window denote the median number of redundant behavioral features represented across observers. Times in brackets indicate the range of each time interval (time started and ended). Beneath, voxels in the rFG show that representational complexity peaks at the top of the rFG in the fourth (183–201 ms) time window (highlighted). Voxel size denotes the number (N) of observers who represented at least one redundant behavioral feature on this voxel and time window. (B) Representation of behavior. Yellow voxels in each time window denote the median MI between MEG activity and the decisions “the Nuns,” “Voltaire,” and “don’t know” across observers (illustrated with the yellow intersection in the Venn diagram). (C) Feature representation for each decision in rFG. Representational complexity was decomposed at each rFG voxel and time window by showing features that are redundantly represented in MEG activity and for each behavioral decision, in each observer (see adjacent color-coded features). Adjacent histograms show the number of rFG voxels representing each redundant feature. The bottom histograms show the median number of voxels representing each redundant feature across observers, showing feature selectivity for each decision (e.g., the turquoise HSF left nuns face and the green LSF bust of Voltaire). See also Figure S4.

If information converges on a brain region to support task behavior, then the number of features represented in the region’s voxels should increase over time—an increase in the complexity of the region’s population code. For each observer, we quantified representational complexity for behavior by counting the number of different features that each brain voxel represents redundantly with behavior, independently in five time intervals over the extended N/M170 time course (see STAR Methods). As shown in Figure 4A, representational complexity does indeed increase over time and peaks between 161–201ms, primarily in the rFG (see Figure S4A for this increase in each observer), the time window during which representation of perceptual decision also peaks in brain activity (see Figures 4B and S4B for each observer). We computed representation of perceptual decision in brain activity as MI < MEG Voxel Activity; “the nuns,” “Voltaire,” “don’t know”>.

Figure 4C decomposes representational complexity into the specific features that underlie each perceptual behavior in each individual observer. In the fourth time window, voxels at the top of the rFG represent redundant features that are linked to the response “Voltaire” (primarily the green global face in special frequency 3 [SF3], the right orange eye in SF1, and the right red eye in SF2). Other redundant features are primarily linked to the response “the nuns” (the turquoise left face in SF1 and the blue and red faces in SF2). Note also that the representation of ipsi-lateral information in the rFG (e.g., the orange and red features) implies that inter-hemispheric information transfer occurs from its initial contra-lateral representation in the left occipital cortex (see Figure 1B and [26]). Figure S4C also shows a trend for HSF features reaching rFG voxels for perceptual behavior before LSF features [27, 28].

Thus, by using feature redundancy and representational complexity, we have demonstrated that rFG voxels represent stimulus information with a selectivity and complexity that supports task-specific behaviors.

## Discussion

In this case study, we investigated how high-dimensional information input collapses in the occipito-ventral pathway to become low dimensional representations that guide behavior, using a novel information theoretic framework called SIR. Using this framework, we identify that high dimensional stimuli are reduced in the occipito-temporal pathway into low dimensional representations that can support subsequent perceptual decision making. To address the where, when, and how of information processing, we tracked dynamic feature representations in the brain and show that behaviorally irrelevant information is rapidly reduced at the occipito-ventral junction around 170 ms. We also show that rFG representations for behavior are constructed between 161 and 201 ms post stimulus. Remarkably, we replicated all these results independently in five individual observers, as is now better practice. Using non-parametric family-wise error rate correction, we found spatio-temporally coincident significant effects within all five observers. This is a stronger finding than conventional cluster corrected group statistics, where a small subset of participants can drive effects that can be non-significant within any individual observer. SIR enabled us to interpret the information processing of task-related brain activity because it computes the interactions between three variables in individual observers (*cf.* the colored set intersections) rather than two of the variables across groups of observers, as is typical in neuroscience and neuroimaging. In doing so, SIR directly addressed the recent argument [29, 30] that neuroscientific explanations need to explicitly include behavior to better tease apart the component processes of the brain.

### Information Reduction in the Occipito-Ventral Pathway

We documented an information reduction process that evolves over time from a state of many to fewer dimensions of stimulus representation. To implement such reduction, hierarchical layers in the occipito-ventral pathway likely communicate with each other, using both feedforward and feedback signals, as suggested by network models that resolve ambiguity between hierarchically organized representations [31, 32]. We subscribe to such interactive organization whereby diagnostic feature selection from the stimulus might involve memory predictions, which propagate down the visual hierarchy and interact with the feedforward flow [5, 33, 34, 35]. Although we can visualize the feedforward flow of stimulus representation by coupling information samples with subsequent brain responses, the arrow of time prevents us from similarly visualizing the representation of top-down predictions (although see [34, 36] for visualizations from behavior). Nevertheless, we can document the interactive architecture by visualizing successive transformations of stimulus representations over time.

We traced the dynamic representation of a nun’s face (the HSF pixels representing this image feature) from occipital cortex into the ventral pathway. It would be naive to assume that the nun’s face is represented as such in any of these regions, but we need a broad view of the information-processing, which this model affords. To better understand representational transformations along the visual hierarchy, we could instead sample an explicit generative model of hierarchical visual information that hypothesize these transformations—with size, rotation, and illumination invariant representations at the top of its hierarchy—to better reflect properties of higher-level ventral pathway representations, and with Gabor-type filters at the bottom, to better model early visual cortex representations [37]. Designing such generative models to study multiple face (e.g., identity, gender, age, ethnicity, and social traits), objects (e.g., superordinate “vehicle,” basic “car,” and subordinate “Beetle”), and scenes (e.g., superordinate “outdoor,” basic “city,” subordinate “Chicago”) categorizations remains a critical step to understand structured sensory representations in wet and silicon brains (*cf* [38, 39, 40, 41]).

### Time Course of Information Processing in the Occipito-Ventral Pathway

The information processes at the occipito-ventral junction flank the timing and sources of the N/M170 ERPs [42], which reflect a network that represents and transfers features across the two hemispheres [15]. Potentially, the N170 peak might reflect the divergence of the two wavefronts of behaviorally relevant and irrelevant information. Alternatively, the pre- and post-170 ms rFG processes could reflect two stages: pre-170 ms, rFG could buffer information arising first from the contra-lateral visual field, followed by ipsi-lateral visual field information that is transferred from the left occipital hemisphere; post-170 ms, rFG could integrate this buffered information across the two visual fields, as shown here (see also [15]). Future research should seek to resolve and generalize these results for the overlapped rFG representations of faces, objects, and scenes categories [43, 44] and elucidate the role of cognitive tasks on representations in pre-frontal cortex [45, 46, 47].

### Relationship between Information Reduction in Occipital Cortex and Consolidation in rFG

Our SIR results inform early versus late attentional models of information selection [48]. We identified where (in occipito-ventral junction) and when (before 170 ms) feature reduction occurs and also where (rFG) and when (from 170 ms) feature are consolidated for perceptual decision. We showed that reduction involves other regions than V1-V2, though these could influence selection with gain control [49, 50]. However, reduction is probably not as late as rFG because this region mainly represents diagnostic features. Our results therefore suggest a mixed model of attentional selection, and SIR offers a powerful platform to directly study such attentional mechanisms in complex tasks.

To conclude, the SIR framework enables us to investigate task-sensitive brain activity that relates information processing in the brain to behavior. SIR enables brain processes to be isolated (here, the reduction of behaviorally irrelevant information and the construction of behavioral representations) and employs principles that are broadly applicable across different modalities and granularities of brain measures used in a wide range of cognitive and systems neuroscience.

## STAR★Methods

### Key Resources Table

**Table:** 

REAGENT or RESOURCE	SOURCE	IDENTIFIER
**Deposited Data**		

Raw and analyzed data	This paper	https://doi.org/10.17632/pjnkwwzn9x.1
Stimuli	This paper	Available upon request

**Software and Algorithms**		

MATLAB R2015b	Mathworks	RRID:SCR_001622
FieldTrip	http://www.fieldtriptoolbox.org/	RRID:SCR_004849
Custom Code (experiment, analyses, visualization)	This paper	Available upon request

### Contact for Reagent and Resource Sharing

Further information and requests for resources should be directed to and will be fulfilled by the Lead Contact, Philippe G. Schyns (philippe.schyns@glasgow.ac.uk).

### Experimental Model and Subject Details

#### Observers

Five right-handed observers with normal (or corrected to normal) vision participated in the experiment. We obtained informed consent from all observers and ethical approval from the University of Glasgow Faculty of Information and Mathematical Sciences Ethics Committee.

### Method Details

#### Stimuli

We cropped a copy of Dali’s *Slave Market with the Disappearing Bust of Voltaire* to retain the ambiguous part of this image that shows the bust of Voltaire and the two nuns. The cropped image size was 256 × 256 pixels, presented at 5.72° × 5.72° of visual angle on a projector screen. On each trial, we sampled information from the cropped image using bubble masks made of randomly placed Gaussian apertures to create a different sparse stimulus. We explain the sampling procedure below (see also Figure 1A-a, Stimulus Sampling). Note that the information supporting perception of “the nuns” and “Voltaire” is separated (i.e., multiplexed [51]) across the spatial frequencies of the stimulus which impact the spatial frequency channels of the visual system (for a review [52],). Consequently, to tap into the information supporting each perception, we decomposed the image into six independent spatial frequency (SF) bands of one octave each, with cut-offs at 128 (22.4), 64 (11.2), 32(5.6), 16 (2.8), 8 (1.4), 4 (0.7) cycles per image (c/deg of visual angle), respectively. For each of the first five SF bands, a bubble mask was generated from a number of randomly located Gaussian apertures (the bubbles), with standard deviations of 0.13, 0.27, 0.54, 1.08, and 2.15 deg of visual angle, respectively. We sampled the image content of each SF band by multiplying the bubble masks and underlying grayscale pixels at that SF band, summed the resulting pixel values across SF bands, and added the constant 6^th^ SF band to generate the actual stimulus image. The total number of 60 Gaussian apertures on each trial remained constant throughout the task, ensuring that equivalent amounts of visual information were presented for each trial, at a level found previously to maintain “don’t know” responses at 25% of the total response number [53]. Since the 6^th^ underlying SF image was constant across trials, we performed all analyses on the 5 bubble masks controlling visibility, but reported only the first three because they represented most of the information required for perceptual decisions. For analysis, we down-sampled (bilinear interpolation) the bubble masks to a resolution of 64 × 64 pixels to speed up computation.

#### Task Procedure

We familiarized each observer with the two possible perceptions of the same stimulus. Each trial started with a fixation cross displayed for 500 ms at the center of the screen, immediately followed by a stimulus generated as explained above that remained until response. We instructed observers to maintain fixation during each trial, and to respond by pressing one of three keys ascribed to each response choice—i.e., “the nuns,” “Voltaire,” or “don’t know.” Each stimulus remained on the screen until response. Stimuli were presented in runs of 150 trials, with randomized inter-trial intervals of 1.5–3.5 s (mean 2 s). Observers performed 4–5 runs in a single day session with short breaks between runs. Observers completed the experiment over 4–5 days.

#### MEG Data Acquisition

We measured the observers’ MEG activity with a 248-magnetometer whole-head system (MAGNES 3600 WH, 4-D Neuroimaging) at a 508 Hz sampling rate. We performed analysis with the FieldTrip toolbox [54] and in-house MATLAB code, according to recommended guidelines [55]. For each observer, we discarded runs based on outlying gradiometer positions in head-space coordinates. That is, we computed the Mahalanobis distance of each sensor position on each run from the distribution of positions of that sensor across all other runs. Runs with high average Malahanobis distance were considered outliers and removed. The distances were then computed again and the selection procedure was repeated until there were no outlier runs (Mahalonobis distances > 20). We high-passed filtered data at 1 Hz (4^th^ order two-pass Butterworth IIR filter), filtered for line noise (notch filter in frequency space) and de-noised via a PCA projection of the reference channels. We identified noisy channels, jumps and other signal artifacts using a combination of automated techniques and visual inspection. We then epoched the resulting dataset (mean trials per observer 3396, range 2885–4154, see Table S1) into trial windows (−0.8 s to 0.8 s around stimulus onset) and decomposed using ICA, separately for each observer. We identified and projected out of the data the ICA sources corresponding to artifacts (eye movements, heartbeat; 3 to 4 components per observer).

We then low-pass filtered the data to 40Hz (3^rd^ order Butterworth IIR filter), specified our interest time period 0-400ms post stimulus, and performed the Linearly Constrained Minimum Variance Beamforming analysis [56] to obtain the source representation of the MEG data on a 6mm uniform grid warped to standardized MNI coordinate space. We low-pass filtered the resulting single trial voxel time courses with a cut-off of 25Hz (3^rd^ order Butterworth IIR filter, two-pass). In the following analysis, based on the obtained single trial voxel activity time courses (12,773 MEG voxels, every 2ms between 0 - 400ms post stimulus), we analyzed the dynamic representation of features in the brain for perceptual decisions.

The following sections detail each step of the information processing pipeline.

### Quantification and Statistical Analysis

#### Diagnostic Features of Behavior

To compute the diagnostic features of perceptual decisions, we quantified the statistical dependence between the pair < Information Samples; Perceptual Decision > using Mutual Information (MI [23]. We used MI because it non-parametrically quantifies the common variations between information and decisions to reveal the features that support decision. On each trial, 5 real-valued Gaussian bubble masks multiply the visual information represented in 5 SF bands (see Figure 1A-a, Stimulus Sampling, for an illustration). Thus, on a given trial, a real value represents the visibility of that pixel under a Gaussian bubble, with 1 indicating full visibility and 0 indicating no visibility. For each pixel of the bubble mask, we converted its random distribution of real values across trials into 2 bins—values below 0.2 were ascribed to the “no to low visibility” bin and values above 0.2 to the “low to full visibility” bin. We then used MI to quantify the statistical dependence between the binarized pixel visibility values and the corresponding observer responses, grouping “the nuns” versus “don’t know” responses together in one computation (i.e., < Information Samples; “the nuns,” “don’t know”>) and the “Voltaire” versus “don’t know” responses in the other (i.e., < Information Samples; “Voltaire,” “don’t know”>). These computations resulted in two MI perceptual decision pixel images per observer (see Figure S1-A). We used the method of maximum statistics [57] to determine the statistical significance of MI pixels and correct for multiple comparisons. Specifically, for each of 10,000 permutations, we randomly shuffled the observer’s choice responses across trials, repeated the computation of MI for each pixel as explained and extracted the maximum MI across all pixels over the 5 SF bands. We used the 99.9^th^ percentile of the distribution of maxes across 10,000 permutations to determine the above-chance significance of each MI pixel (FWER p < 0.001, one-tailed). Across observers, we reported the diagnostic pixels with significant MI in the first 3 SF bands that illustrate the consistency of the main diagnostic features underlying perceptual decision behaviors (see Figure 1A-c, Diagnostic Features of Behavior).

#### Brain Features

In each observer, we measured single-trial MEG activity with the bivariate of amplitude and instantaneous MEG gradient on 12,773 sources, every 2 ms between 0 and 400 ms post stimulus. A high-dimensional 12,773 × 200 voxel-by-time matrix therefore structures the MEG data. For each observer, we aimed to quantify the features of the stimulus that each cell of this matrix represents, if any. We proceeded in three steps. We now detail the computations involved in each step. For MI calculations, we used throughout the Gaussian-Copula Mutual Information estimator [23]. Note that we report only the 5,869 cortical voxels in our figures.

##### Step 1: Computation of the Relationship < Information Samples; MEG Activity >

We aim to identify, in each observer, the features represented in each cell of the full voxel-by-time matrix of MEG activity. However, it is computationally impractical to directly compute the features from the single-trial relationship < Information Samples; MEG Voxel Activity >, due to the enormous dimensionality of the space—64 × 64 × 5 SF bands pixels x 12,773 voxels x 200 time points. Instead, we used the method reported in [26], which computes the relationship over the more computationally tractable matrix of 60 Independent Component Analysis (ICA) sources representing MEG activity over 75 time points that span 0 to 600 ms post stimulus every 8 ms.

##### Step 2: Computation of Brain Features

For each observer, the reduced matrix computed above (i.e., 60 ICA sources x 75 time points) comprised MI images in each cell, for a total of 4,500 MEG-pixel information images across 5 SF bands. We vectorized each (64 × 64 × 5 = 200,480) MEG MI image as a 20,480-dimensional vector. We applied Non-negative Matrix Factorization (NMF [58],) to the set of 4,500 vectorized MEG MI images to characterize the main NMF features of the stimulus that modulate MEG source activity, resulting in 21–25 components per observer. We thresholded these NMF features by setting to zero the pixels with low MI values (< 15% of the maximum pixel value across SFs). We then normalized the NMF features (L2-norm). Henceforth, we call “brain features” the normalized NMF features of each observer that modulate the MEG activity of their brain.

##### Step 3: Computation of the Relationship < Brain Feature; MEG Voxel Activity > in the Full Voxel-by-Time MEG Activity Matrix

We used the brain features computed above from the reduced matrix of ICA MEG sources to quantify their representation into each cell of the full voxel-by-time matrix. To this aim, first we computed the visibility of each brain feature into the information samples (i.e., bubble mask) presented as stimulus on each trial. That is, we spatially filtered (i.e., dot product) the bubble mask for that trial with the brain feature computed above, thereby producing a scalar value indicating the visibility of this feature on this trial. We call these real values “brain feature coefficients.” Next, for each brain feature, and for each cell of the full voxel-by-time MEG activity matrix, with MI we quantified the relationship < Brain Feature Coefficient; MEG Voxel Activity >. This produced for each observer, a 3D feature-by-voxel-by-time MI matrix. We determined the statistical significance for each cell using a permutation approach and the method of maximum statistic to address multiple comparisons [57]. Specifically, for each of 200 permutations, we randomly shuffled the brain feature coefficients values across trials and recalculated the MI of the single trial relationship < Randomized Brain Feature Coefficients; MEG Voxel Activity >. We then computed the maximum of the resulting 3D MI matrix for each permutation and used the 95^th^ percentile of this maximum value across permutations as the statistical threshold (i.e., FWER p < 0.05, one-tailed). In the remaining analyses, we used the thresholded 3D feature-by-voxel-by-time MI matrix of each observer (called “representation matrix” in the main text).

#### Diagnostic and Nondiagnostic Brain Features

For each observer, we determined the diagnostic versus nondiagnostic status of their brain features as follows. Using only the trials associated with “the nuns” versus “don’t know” responses, we computed the single-trial MI relationship < Brain Feature Coefficient; “the nuns,” “don’t know”>. We computed independently the single-trial MI relationship < Brain Feature Coefficient; “Voltaire,” “don’t know”>, using these trials. In both cases, a strong relationship (i.e., MI above 75^th^ percentile of the distribution of MI across all brain features) would classify this brain feature as diagnostic—i.e., of “the nuns” or of “Voltaire.” Finally, we computed the single-trial MI relationship < Brain Feature Coefficient; “the nuns,” “Voltaire,” “don’t know”>. A weak relationship (MI below 25^th^ percentile of the MI distribution) would classify this brain feature as nondiagnostic of perceptual decisions (see Figure S1-B for the perception-specific brain features and nondiagnostic features of each observer).

#### K-means of Brain Features

Observers’ brains represented similar brain features in the task (see Figure S1B). This warranted their projection onto a common feature basis for group-level visualization. To this aim, we applied k-means clustering by setting *k*, the number of clusters, to 25, to align the number of means to the maximum number of NNMF brain features computed in any observer. We pooled the normalized NNMF brain features of all observers, resulting in a 115 × 20480 matrix (115 NNMF components in total for 5 observers and 64 pixels ^∗^ 64 pixels ^∗^ 5 SFs weights). We applied k-means (cosine similarity, 1000 repetitions) to this matrix. It is important to emphasize that we performed all analyses on the specific brain features of each observer. We only indexed these individual features onto the common k-mean feature basis and corresponding color codes to report group results (e.g., in Figure 1 and 4).

#### Divergence of Brain Features

For each observer, we used their full 3D un-thresholded 3D representation matrix. For each of the 5,869 cortical voxels, we extracted the max MI across all diagnostic (versus nondiagnostic) features in 10 ms time windows between 0 and 400 ms post stimulus. This resulted in one 2D matrix (5869 voxels by 40 time windows) of diagnostic feature representation and another of nondiagnostric feature representation. In each time window, we computed the similarity between diagnostic and nondiagnostic representations with the de-meaned dot-product between the two 5,869 dimensional vectors. To establish statistical significance, we bootstrapped a null distribution as follows. On each iteration (N = 1000), we randomly shuffled the values across the dimensions of the two 5,869 dimensional vectors and calculated their de-meaned dot product. We used the percentile 0.625 and 99.9375 of the chance distribution as the upper and lower boundaries for the chance-level similarity (Bonferroni corrected, p < 0.05, 2-tailed). We performed the same analysis at the group level, by pooling all participant’s data together to form a larger 2D matrix (29345 voxels by 40 time windows). We found diagnostic and nondiagnostic brain features diverge around 170 ms post stimulus (see Figure S2-B).

On this basis, we defined an earlier ([50-170 ms] post stimulus) and a later time window ([170-400 ms] post stimulus) and summarized the representation of brain features in each. A voxel would represent diagnostic (versus nondiagnostic) brain features if it has significant MI (FWER p < 0.05, one-tailed) for at least one diagnostic (versus nondiagnostic) brain feature in this time window. For each voxel, we then counted the number of observers satisfying these criteria and reported the distributions for diagnostic (white schematic brains in Figure 2) and nondiagnostic (magenta schematic brains in Figure 2) brain features in each time window.

#### Dynamic Feature Representation in Occipital Cortex

For each observer, we proceeded in two steps:

##### Step 1: Dynamics of brain feature representation between 0 and 400ms post stimulus

For each observer, we used their representation matrix and selected the voxels with significant MI for at least one nondiagnostic brain feature in the 0 to 400ms time window (henceforth, “nondiagnostic voxels”). For each nondiagnostic voxel, at each time point, we extracted the maximum MI over all nondiagnostic brain features to plot the maximum representation curve of this voxel (Figure 3A shows the representation curves of all nondiagnostic voxels). The curve of each voxel had an onset (the first time point at which maximum MI was significant) and an offset (the last time point of significance) that we computed; representation duration on a voxel was therefore computed as offset - onset. Finally, we computed the Euclidean distance (in the common MNI space) of each voxel in relation to the voxel with the earliest onset. We repeated these computations separately for diagnostic brain features.

For nondiagnostic voxels (versus diagnostic voxels), we fitted a robust linear regression line between their onset times and Euclidean distances from the voxel of initial onset. We computed another robust linear regression between their representation duration and Euclidean distances (see Figure S3 for individual results). Table S2 details the statistics of the robust linear regressions. We excluded outlier voxels for these analyses—i.e., voxels with > 3 standard deviations from the median onset of all voxels, computed separately using nondiagnostic and diagnostic voxel onset distributions, see Table S3 for percentage of voxels exclusion.

##### Step 2: Spatial-temporal junction of divergence between nondiagnostic and diagnostic feature representations

We selected the voxels representing nondiagnostic features that were furthest in the brain–i.e., with Euclidean distances > 75^th^ percentile of distances of all nondiagnostic voxels. These voxels represented the spatial marker of the junction. We defined the latest representation offsets of these voxels as the temporal marker of the junction (see Figure 3A the vertical dash line on the representation curves). To identify the brain regions (based on the “Talairach Demon Atlas” warped into MNI space) involved in the junction, we grouped nondiagnostic voxels of each observer according to their location in the cuneus (CU), lingual gyrus (LG), inferior occipital cortex (IOG), middle occipital gyrus (MOG), superior occipital gyrus (SOG), fusiform gyrus voxels locates quite close to LG (LG/FG, see Figure S3-D for location), fusiform gyrus (FG), inferior temporal gyrus (ITG), middle temporal gyrus (MTG), superior temporal gyrus (STG), inferior parietal lobe (IPL), and superior parietal lobe (SPL). In each anatomical region, we then checked the Euclidean distance (see step 1) of all nondiagnostic and diagnostic voxels (see Figure 3C and Figure S3-C for individual results).

#### Feature Representation in the Brain for Behavior

For each observer, we proceeded in three steps:

##### Step 1: Redundancy computation: < Brain Feature Coefficients; MEG Voxel Activity; Perceptual Decision >

For each observer, we computed information theoretic redundancy, from co-information [23], the triple relationship between < Brain Feature Coefficients; MEG Voxel Activity; “the nuns,“ “Voltaire,” “don’t know”>:

(1) RED=MI(Feature;PerceptualDecision)+MI(Feature;MEGVoxelActivity)−MI(Feature;MEGVoxelActivity,PerceptualDecision)

(1) is equivalent to the set theoretic intersection of three variable entropies, or alternatively the intersection of any two mutual information [23]. We applied Equation (1) for each combination of diagnostic brain feature, brain voxel, and every 2 ms between 0 and 400 ms post stimulus. This computation produced a 3D redundancy matrix (feature × voxel × time point). We established statistical significance for each cell by recomputing redundancy with shuffled decision responses across trials (repeated 200 times), and used the 95^th^ percentile of 200 maximum values (each taken across of the entire 3D redundancy matrix per permutation) as statistical threshold (i.e., FWER, p < 0.05, one-tailed)

##### Step 2: Representational complexity computation

We constructed 5 evenly distributed time windows per observer between 120 and 220 ms. This specific time interval encompasses the M/N170 time course. In each time window, for each of the 12,773 brain voxels, we calculated the median number of different redundant features it significantly represented across five observers (see gray level scatters in Figure S4-A).

##### Step 3: Representation of behavior in the brain

For each voxel, we also computed MI < MEG Voxel activity, “the nuns,” “Voltaire,” “don’t know” >, resulting in a 2D voxel by time matrix. To establish statistical significance, we extracted the maximum MI across the matrix recomputed, shuffling decision responses across trials in each cell (repeated 200 times). We used the 95^th^ percentile of this maximum distribution as statistical threshold (i.e., FWER, p < 0.05, one-tailed). In each time window (see Step 2), for each brain voxel we calculated the median MI value (see orange scatters in Figure S4-B).

##### Step 4: Decision-specific feature representations

We uncovered the perception-specific redundant features of each observer by computing information theoretic redundancy between < Brain Feature Coefficients; MEG Voxel Activity; “the nuns,“ “don’t know”>, and between < Brain Feature Coefficients; MEG Voxel Activity; “Voltaire,“ “don’t know”>, separately. We used the permutation test described in Step 1 above to threshold redundancy values and obtain the features represented on rFG voxels for each perceptual decision behavior (see color-coded scatters in Figure S4-C for each observer).

### Data and Software Availability

The raw data and analyzed data reported in this study are deposited in Mendeley Data: https://doi.org/10.17632/pjnkwwzn9x.1. The custom code (experiment, analyses, visualization) are available by request to the Lead Contact.

## Supplemental Information

Document S1. Figures S1–S4 and Tables S1–S3

Document S2. Article plus Supplemental Information
