# Supplementary material for: Dynamic Construction of Reduced Representations in the Brain for Perceptual Decision Behavior
Source: Curr Biol. 2019 Jan 21;29(2):319–326.e4. doi: 10.1016/j.cub.2018.11.049 (PMC6345582; doi:10.1016/j.cub.2018.11.049)
Supplement: Document S1. Figures S1–S4 and Tables S1–S3 [file mmc1.pdf]

Current Biology, Volume *29*

Supplemental Information

Dynamic Construction of Reduced Representations in the Brain for Perceptual Decision Behavior

Jiayu Zhan, Robin A.A. Ince, Nicola van Rijsbergen, and Philippe G. Schyns

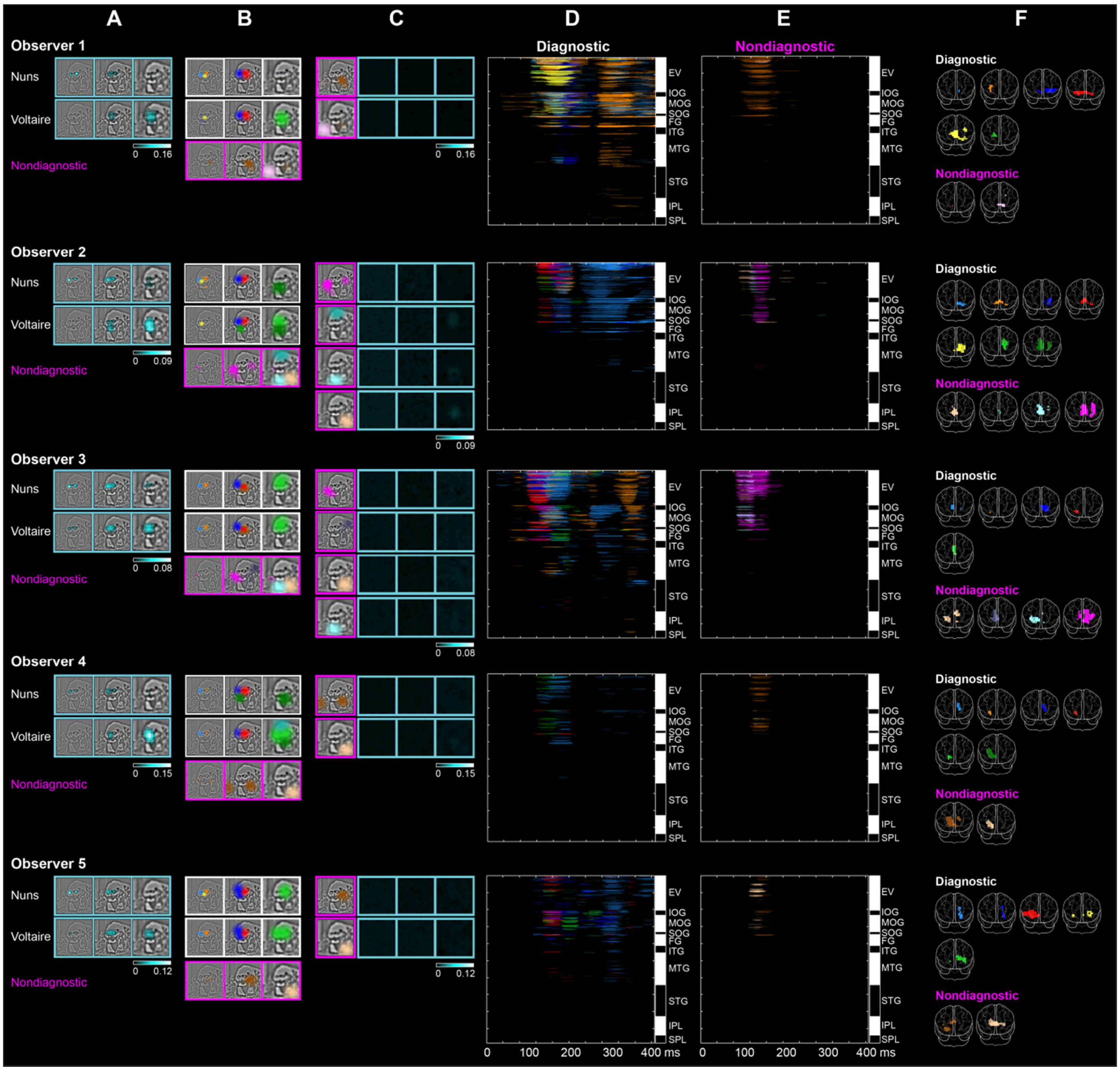

Figure S1. Diagnostic Features and Brain Features of each Observer. Related to Figure 1.

A. Diagnostic Features. The cyan framed images in column A report the significant (FWER $p < 0.01$, one-tailed) MI value for each pixel in the first three spatial frequency (SF) bands, revealing across observers the features most diagnostic for responding "the nuns" (the two small faces in SF band 1) and "Voltaire" (the broad face in SF band 3). B. Brain Features. White frames in column B (vs. magenta frames in column C) highlight the diagnostic (vs. nondiagnostic) features that this observer's MEG voxels represented, separately presented for deciding "the nuns" and "Voltaire." C. Nondiagnostic features Influence Brain Measures but not Behavior. We computed whether the presence of background nondiagnostic features (magenta frames in column C) modulated the usage of the foreground diagnostic features (column A) for perceptual decisions. We computed the *Modulation Index = MI <Information Samples, Perceptual Decision> - CMI <Information Samples, Perceptual Decision | Nondiagnostic Feature>*. The first term (Mutual Information, MI), computes the relationship between all information samples and perceptual decisions—i.e. the diagnostic features. The second term (CMI) recomputes this relationship, eliminating (i.e. conditioning out) the role of background nondiagnostic features (each magenta frame in column C). The difference is a

modulation index image (cyan framed per spatial frequency in column C), in which values indicates whether nondiagnostic features change the usage of diagnostic features—as can be seen in the cyan frames of column C, they did not (see Figure S2A for the statistical demonstration). D and E. Maximum Diagnostic and Nondiagnostic Representation Matrices. Each cell of the Diagnostic (column D) and Nondiagnostic (column E) representation matrices report the color-coded significant brain feature with maximum representation in MEG effect size (i.e. MI) across all brain features, at this voxel and time point. For reference, alternating white/black bars flanking each matrix indicate the anatomical brain region of the corresponding voxels. To illustrate, the representation matrices of Observer 1 reveal that the diagnostic brain feature “nose of Voltaire” in yellow is primarily represented in specific EV, MOG and FG voxels with highest effect sizes across the full-time course. In contrast, the brown nondiagnostic brain feature is primarily represented in occipital regions, and before ~170 ms. F. Early Representation of Brain Features. Color-coded brain regions show the early (i.e. initial 20 ms) topological representation of diagnostic and nondiagnostic brain features (FWER, $p < 0.05$, one-tailed). EV = early visual cortex (including lingual gyrus and cuneus), IOG = inferior occipital gyrus, MOG = middle occipital gyrus, SOG = superior occipital gyrus, FG = fusiform gyrus, ITG = inferior temporal gyrus, MTG = middle temporal gyrus, STG = superior temporal gyrus, IPL = inferior parietal lobe, SPL = superior parietal lobe.

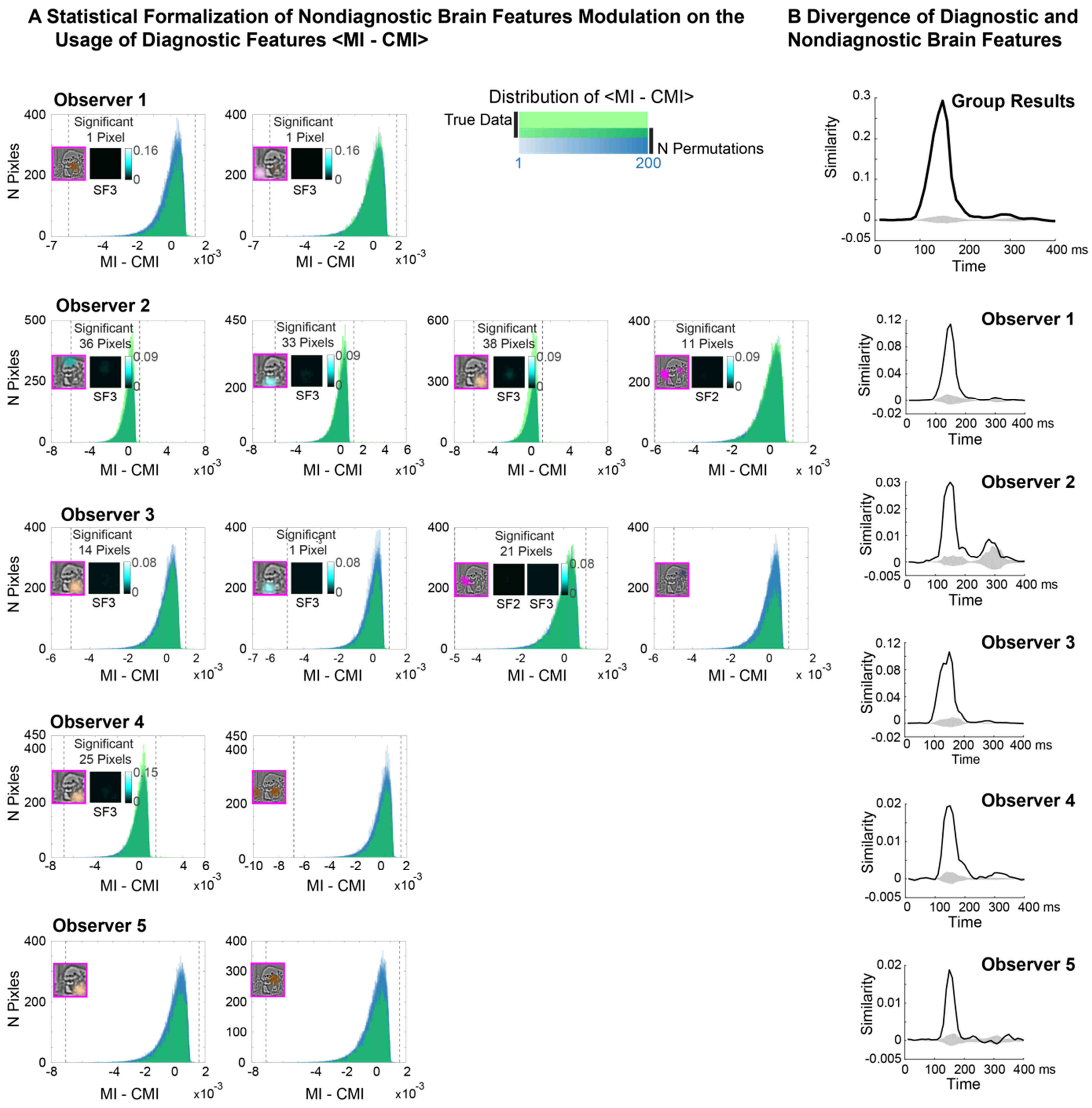

Figure S2. Divergence of Diagnostic Nondiagnostic Brain Features. Related to Figure 2 and STAR METHOD, QUANTIFICATION AND STATISTICAL ANALYSIS, Divergence of Brain Features.

<u>A. Statistical Testing of the Modulation Index.</u> In each observer (rows), each panel displays the green distribution of the number of pixels (Y axis) with modulation index values (X axis, MI - CMI) for a given nondiagnostic feature (magenta insert). The blue distribution shows chance-level modulations, obtained by first shuffling nondiagnostic feature coefficients across trials and then recalculating CMI in each of 200 permutations. A shade of blue illustrates the high overlap between the true data and permutated chance distributions. The left and right dashed lines indicate the lower vs. upper significance thresholds (p < .05, two-tailed, corrected with the methods of maximum statistics). The small number of pixels significantly modulated by nondiagnostic features are shown in a frame adjacent to the nondiagnostic feature for their number, image location(s) and number. <u>B. Divergence of</u>

Diagnostic and Nondiagnostic Brain Features. Group Results plot shows the similarity between diagnostic and nondiagnostic brain feature representations over the time course of visual information processing, using the data of all observers. The shadowed region indicates the Bonferroni corrected chance-level similarity ($p < 0.05$, two-tailed). Observer 1 to 5 plots show the diagnostic vs. nondiagnostic brain feature representation similarity for each observer. Together, the results show a consistent dynamic pattern of increasing similarity of diagnostic and nondiagnostic feature representations in the brain of each observer, up until 170 ms post-stimulus, following which diagnostic and nondiagnostic feature representations become dissimilar (i.e. diverge).

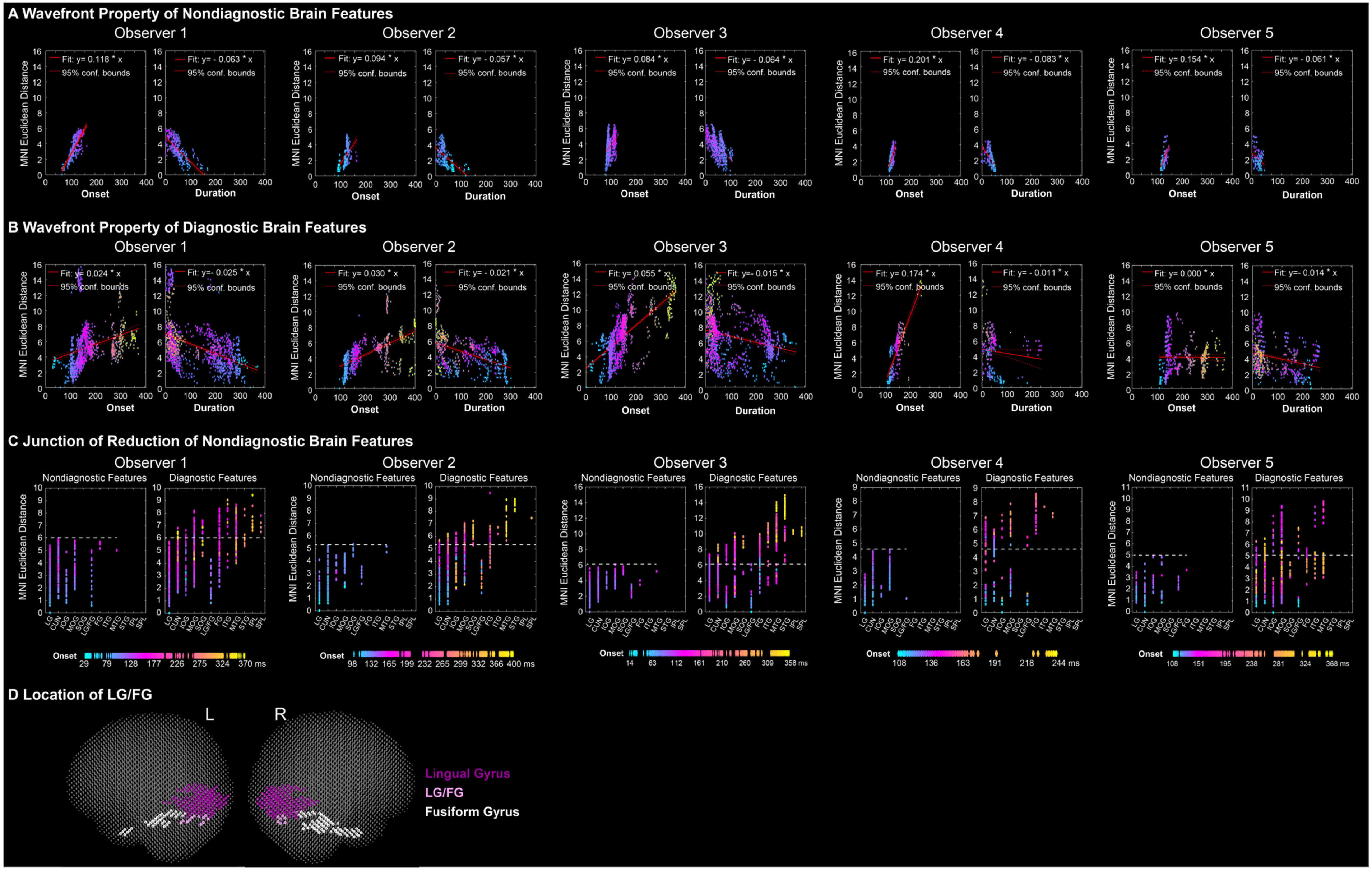

A Wavefront Property of Nondiagnostic Brain Features
Observer 1
Observer 2
Observer 3
Observer 4
Observer 5
MNI Euclidean Distance
Onset
Duration
Fit: y= 0.118 * x
Fit: y= - 0.063 * x
Fit: y= 0.094 * x
Fit: y= - 0.057 * x
Fit: y= 0.084 * x
Fit: y= - 0.064 * x
Fit: y= 0.201 * x
Fit: y= - 0.083 * x
Fit: y= 0.154 * x
Fit: y= - 0.061 * x
95% conf. bounds
B Wavefront Property of Diagnostic Brain Features
Fit: y= 0.024 * x
Fit: y= - 0.025 * x
Fit: y= 0.030 * x
Fit: y= - 0.021 * x
Fit: y= 0.055 * x
Fit: y= - 0.015 * x
Fit: y= 0.174 * x
Fit: y= - 0.011 * x
Fit: y= 0.000 * x
Fit: y= - 0.014 * x
C Junction of Reduction of Nondiagnostic Brain Features
Nondiagnostic Features
Diagnostic Features
LG CUN IOG MOG SOG LG/FG FG ITG MTG STG IPL SPL
Onset 29 79 128 177 226 275 324 370 ms
Onset 98 132 165 199 232 265 299 332 366 400 ms
Onset 14 63 112 161 210 260 309 358 ms
Onset 108 136 163 191 218 244 ms
Onset 108 151 195 238 281 324 368 ms
D Location of LG/FG
L
R
Lingual Gyrus
LG/FG
Fusiform Gyrus

Figure S3. Dynamic Reduction of Nondiagnostic Brain Feature Representations in Occipito-Ventral Pathway of Each Observer. Related to Figure 3.

A. Wavefront Property of Nondiagnostic Brain Feature Representations. The left scatter shows a linear relation between the representation onset times of voxels and their Euclidean distances to the voxel of initial representation onset. The right scatter shows that duration of nondiagnostic feature representation linearly decreases with the increasing distance of the considered voxel from the voxel of initial representation onset. B. Wavefront Property of Diagnostic Brain Feature Representation. Same caption as in panel A for diagnostic brain features, with later onsets (pink to yellow colors) in ventral and dorsal regions. C. Junction of Reduction of Nondiagnostic Brain Features. In the left panel, voxels color-coded by onset times are pooled by anatomical brain region (X-axis) and scattered according to their Euclidean distance to the initial onset voxel of nondiagnostic representation on the Y-axis. In the right panel, the same caption for diagnostic voxels. The horizontal dashed line indicates the brain regions of furthest representation of nondiagnostic features. LG/FG on the X-axis comprises voxels located near to LG, which are illustrated in panel D. In D, the dark purple scatters show lingual gyrus (LG) voxels; the light purple scatters show LG/FG voxels which are fusiform gyrus voxels located next to lingual gyrus voxels; the white scatters show the well-demarcated FG voxels that we included in our analysis of feature representations for behavior. LG = Lingual Gyrus, CUN = cuneus, IOG = inferior occipital gyrus, MOG = middle occipital gyrus, SOG = superior occipital gyrus, FG = fusiform gyrus, ITG = inferior temporal gyrus, MTG = middle temporal gyrus, STG = superior temporal gyrus, IPL = inferior parietal lobe, SPL = superior parietal lobe.

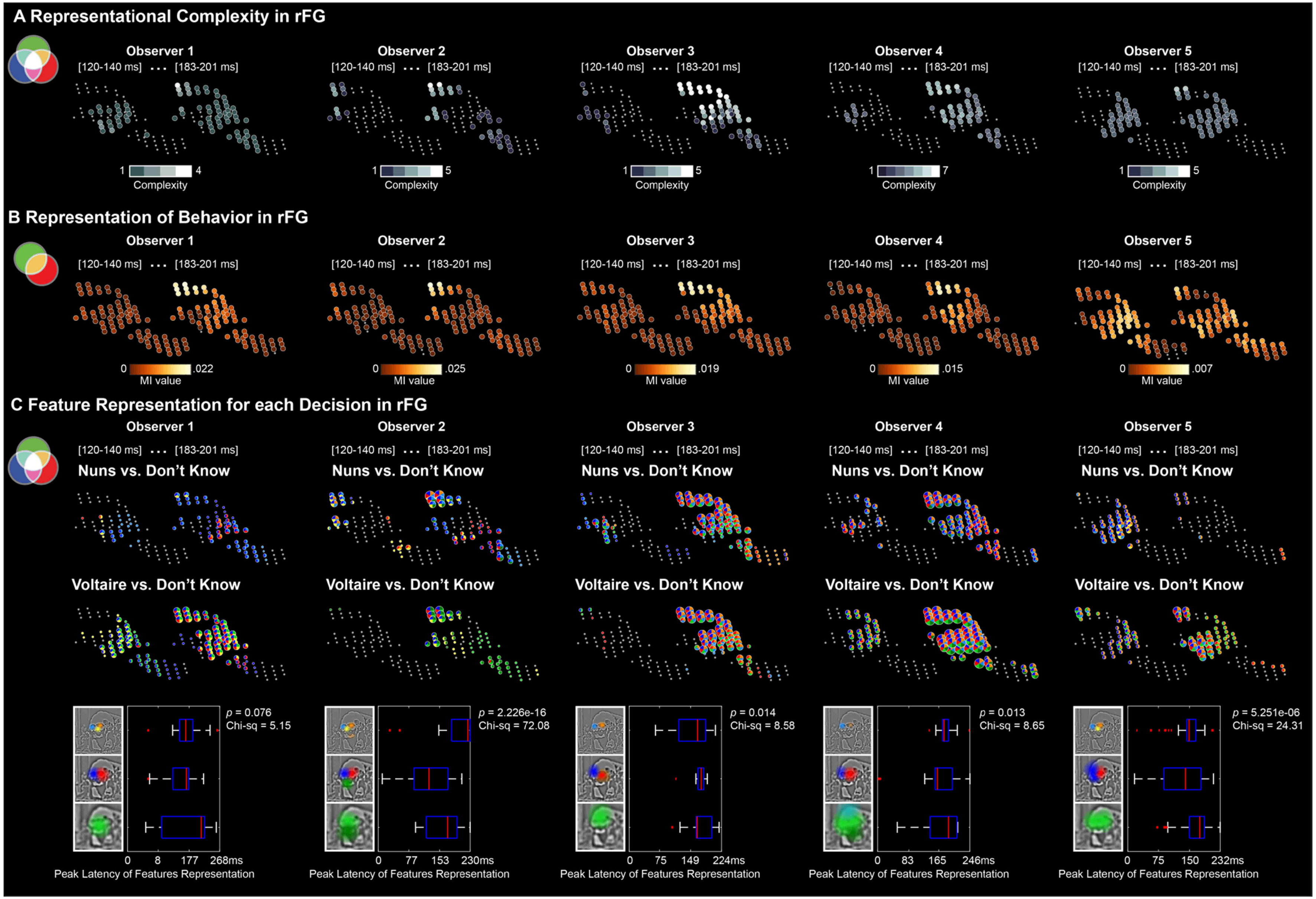
A Representational Complexity in rFG
Observer 1
[120-140 ms] ... [183-201 ms]
1 Complexity 4
Observer 2
[120-140 ms] ... [183-201 ms]
1 Complexity 5
Observer 3
[120-140 ms] ... [183-201 ms]
1 Complexity 5
Observer 4
[120-140 ms] ... [183-201 ms]
1 Complexity 7
Observer 5
[120-140 ms] ... [183-201 ms]
1 Complexity 5
B Representation of Behavior in rFG
Observer 1
[120-140 ms] ... [183-201 ms]
0 MI value .022
Observer 2
[120-140 ms] ... [183-201 ms]
0 MI value .025
Observer 3
[120-140 ms] ... [183-201 ms]
0 MI value .019
Observer 4
[120-140 ms] ... [183-201 ms]
0 MI value .015
Observer 5
[120-140 ms] ... [183-201 ms]
0 MI value .007
C Feature Representation for each Decision in rFG
Observer 1
[120-140 ms] ... [183-201 ms]
Nuns vs. Don't Know
Voltaire vs. Don't Know
p = 0.076
Chi-sq = 5.15
0 8 177 268ms
Peak Latency of Features Representation
Observer 2
[120-140 ms] ... [183-201 ms]
Nuns vs. Don't Know
Voltaire vs. Don't Know
p = 2.226e-16
Chi-sq = 72.08
0 77 153 230ms
Peak Latency of Features Representation
Observer 3
[120-140 ms] ... [183-201 ms]
Nuns vs. Don't Know
Voltaire vs. Don't Know
p = 0.014
Chi-sq = 8.58
0 75 149 224ms
Peak Latency of Features Representation
Observer 4
[120-140 ms] ... [183-201 ms]
Nuns vs. Don't Know
Voltaire vs. Don't Know
p = 0.013
Chi-sq = 8.65
0 83 165 246ms
Peak Latency of Features Representation
Observer 5
[120-140 ms] ... [183-201 ms]
Nuns vs. Don't Know
Voltaire vs. Don't Know
p = 5.251e-06
Chi-sq = 24.31
0 75 150 232ms
Peak Latency of Features Representation

Figure S4. Dynamic Construction of Representations for Behavior in rFG of each Observer. Related to Figure 4.

A. Representational Complexity in rFG. Starting and ending times in brackets indicate the ranges of the time intervals we displayed for this observer. Here, we only show two critical time windows (1st and 4th in Figure 4). The grey level of the right Fusiform Gyrus (rFG) voxels corresponds to the number of redundant features that it represented within each time interval. B. Representation of Behavior in rFG. Yellow voxels denote the maximum MI (un-thresholded) between MEG activity and decisions "the Nuns", "Voltaire," "don't know" in each time interval. The yellow level represents the maximum MI value. C. Feature Representation for each Decision. Specific redundant features represented at each rFG voxel and time interval for each decision behavior (see the color-coded features for interpretation). The bottom box-plots show the peak latency of redundant feature representations (Y-axis) across rFG voxels at each Spatial Frequency (SF) band over time (X-axis). We used the representation matrices to compute the peak representation latency (i.e. peak MI) of each redundant feature on each rFG voxel. We computed the median peak latency of all redundant features per SF band and compared peak latencies across SF bands with a non-parametric one-way ANOVA (i.e. Kruskal-Walis test). Comparison of median peak latencies revealed that higher SF features tend to be represented on rFG voxels before lower SF features, a result compatible with [S1, S2].

Observer	All responses	"Nuns" response	"Voltaire" response	"Don't Know" response
1	3314	1189	1313	812
2	3604	1666	1263	675
3	4154	1634	1892	628
4	3023	1603	738	682
5	2885	1007	1346	532

Table S1. Number of trials following pre-processing of MEG data. Relate to STAR METHODS, METHOD DETAILS, MEG Data Acquisition.

**Nondiagnostic voxels**				
	Onset		Duration	
Observer	model	p value	model	p value
1	Y=0.118X - 3.546	p < .001	Y = -0.063X + 4.906	p < .001
2	Y=0.094X - 3.222	p < .001	Y = -0.057X + 3.673	p < .001
3	Y=0.084X -0.457	p < .001	Y = -0.064X + 5.189	p < .001
4	Y=0.201X - 10.535	p < .001	Y = -0.083X + 3.746	p < .001
5	Y=0.154X - 7.912	p < .001	Y = -0.061X + 2.869	p < .001
**Diagnostic voxels**				
	Onset		Duration	
Observer	model	p value	model	p value
1	Y=0.024X + 3.214	p < .001	Y = -0.025X + 7.013	p < .001
2	Y=0.030X + 1.343	p < .001	Y = -0.021X + 5.852	p < .001
3	Y=0.055X + 2.462	p < .001	Y = -0.015X + 7.408	p < .001
4	Y=0.174X - 8.126	p < .001	Y = -0.011X + 5.004	p = .094
5	Y=0.000X + 4.061	p = .919	Y = -0.014X + 4.693	p < .001

Table S2. Linear models between the Euclidean distance (Y) and Onset/Duration (X), and p values for the slope, per observer. Relate to Figure 3 and STAR METHODS, QUANTIFICATION AND STATISTICAL ANALYSIS, Dynamic Feature Representation in Occipital Cortex.

Observer	Diagnostic	Nondiagnostic
1	1.81%	0.35%
2	0	2.45%
3	2.21%	0.19%
4	3.52%	0
5	0.58%	0

Table S3. Percentage of voxels excluded from onset analysis. Relate to STAR METHODS, QUANTIFICATION AND STATISTICAL ANALYSIS, Dynamic Feature Representation in Occipital Cortex.

Supplemental References

S1. Schyns, P.G., Petro, L.S., and Smith, M.L. (2009). Transmission of Facial Expressions of Emotion Co-Evolved with Their Efficient Decoding in the Brain: Behavioral and Brain Evidence. Plos One *4,* e5625.

S2. van Rijsbergen, N.J., and Schyns, P.G. (2009). Dynamics of trimming the content of face representations for categorization in the brain. Plos Comput Biol *5*, e1000561.
